# Pancreaticoduodenectomy for a primary duodenal capicua transcriptional repressor (CIC) -rearranged sarcoma with severe bleeding: a case report

**DOI:** 10.1186/s12876-020-01266-3

**Published:** 2020-04-15

**Authors:** Yuichi Aoki, Hisashi Oshiro, Akihiko Yoshida, Kazue Morishima, Atsushi Miki, Hideki Sasanuma, Yasunaru Sakuma, Alan Kawarai Lefor, Naohiro Sata

**Affiliations:** 1 Division of Gastroenterological, Department of Surgery, General and Transplant Surgery, Jichi Medical University, 3311-1, Tochigi, Yakushiji Shimotsuke, 329-0498 Japan; 2 Department of Pathology, Jichi Medical University, 3311-1, Tochigi, Yakushiji Shimotsuke, 329-0498 Japan; 3 Department of Diagnostic Pathology, National Cancer Center Hospital, Tokyo, Japan

**Keywords:** CIC-rearranged sarcoma, Duodenum, Ewing-like sarcoma, Ewing sarcoma, Oncologic emergency, Gastrointestinal bleeding, Pancreaticoduodenectomy

## Abstract

**Background:**

Capicua transcriptional repressor (CIC) -rearranged sarcoma is characterized by small round cells, histologically similar to Ewing sarcoma. However, CIC-rearranged sarcoma has different clinical, histological, and immunohistochemical features from Ewing sarcoma. It is important to differentiate between these tumors.

**Case presentation:**

The patient is a 44-year-old man with a duodenal tumor diagnosed in another hospital who presented with a history of melena. Laboratory studies showed anemia with a serum hemoglobin of 6.0 g/dL. He was hospitalized and gastrointestinal bleeding was controlled successfully with endoscopy. However, he suffered from appetite loss and vomiting and progression of anemia a few weeks after presentation. Upper gastrointestinal endoscopy showed a circumferential soft tumor in the second portion of the duodenum and the endoscope could not pass distally. Computed tomography scan showed a greater than 10 cm tumor in the duodenum, with compression of the inferior vena cava and infiltrating the ascending colon. A definitive pathologic diagnosis could not be established despite four biopsies from the tumor edge. Due to gastrointestinal obstruction and progression of anemia, a pylorus-preserving pancreaticoduodenectomy with partial resection of the inferior vena cava and right hemicolectomy was performed as a complete tumor resection. The tumor was diagnosed as a CIC-rearranged sarcoma, but 2 months postoperatively local recurrence and distant metastases to the liver and lung were found. The patient died 3 months after surgery.

**Conclusions:**

Although the only definitive treatment for CIC-rearranged sarcoma is surgical resection, the CIC-rearranged sarcoma is highly malignant with a poor prognosis even after radical resection. More research is needed to establish optimal treatment strategies.

## Background

In recent years, a disease concept called ‘capicua transcriptional repressor (CIC) -rearranged sarcoma’ with gene rearrangement has been recognized [1]. CIC-rearranged sarcoma is small round cell tumor, histologically similar to Ewing sarcoma, but without a Ewing sarcoma breakpoint - est. variant gene (EWSR-ETS) and show no translocation. CIC-rearranged sarcoma was included in the group of ‘Ewing-like sarcomas’ in the past. CIC-rearranged sarcomas have different clinical, histological, and immunohistochemical features from Ewing sarcoma. Although we must differentiate between these two tumors, it is difficult because of their rarity.

We present a patient with a primary CIC-rearranged sarcoma of the duodenum with severe gastrointestinal bleeding treated with pancreaticoduodenectomy, partial resection of the inferior vena cava and right hemicolectomy.

## Case presentation

The patient is a 44-year-old man who presented with a duodenal tumor recently diagnosed at an outside facility. He was admitted to another hospital with melena and anemia with a serum hemoglobin of 6.0 g/dL. Four units of red cell concentrate was transfused and upper gastrointestinal endoscopy performed for hemostasis of the duodenal lesion. Gastrointestinal bleeding was then stabilized for several days. The patient was discharged from that hospital and returned home with instructions to be evaluated at our hospital. Two weeks later, he presented with appetite loss and vomiting. Past medical history, family history and social history were unremarkable. The physical examination was unremarkable. Laboratory studies showed a serum hemoglobin of 7.8 g/dL. Serum tumor marker levels were within normal limits (CEA 0.7 ng/ml, CA19–9 7 U/ml, IL-2 receptor 323 U/ml).

**Fig. 1:**
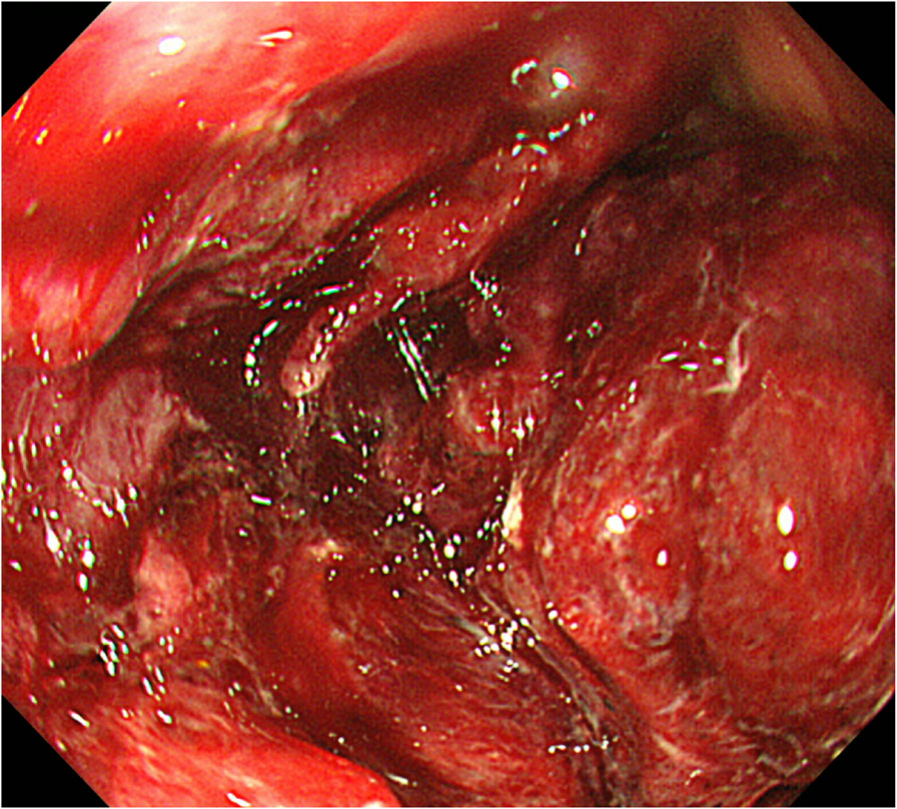
Upper gastrointestinal endoscopy showed a circumferential soft tumor with a friable surface in the second portion of the duodenum. It was difficult to pass the endoscope distally and the papilla could not be identified

Repeat upper gastrointestinal endoscopy showed a circumferential soft tumor with a friable surface in the second portion of the duodenum. It was difficult to insert the endoscope distally, and difficult to identify the papilla (Fig. 1). Though four biopsies were taken from the tumor edge and a small round cell sarcoma was suspected, a definitive diagnosis could not be made.

**Fig. 2:**
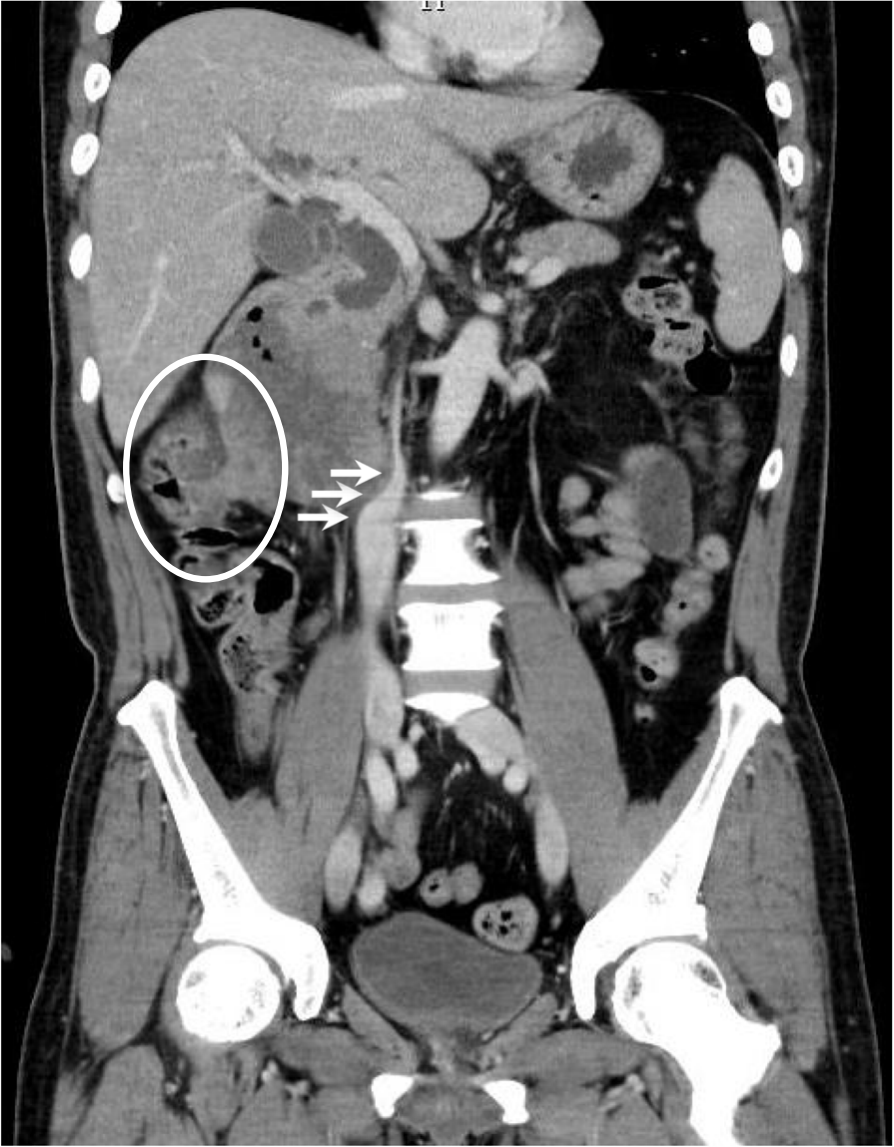
Computed tomography scan showed a greater than 10 cm tumor with heterogeneous enhancement. The central portion contained air suggesting necrosis and infection. No obvious metastases were seen in other abdominal viscera lung fields, and intraperitoneal lymph nodes were not enlarged. Arrows point to the compressed inferior vena cava. The circle shows infiltration to the ascending colon

Computed tomography (CT) scan showed a greater than 10 cm tumor with heterogeneous enhancement on a dynamic study and some air suggesting necrosis and infection inside the tumor (Fig. 2). The tumor compressed the inferior vena cava and infiltrated the ascending colon. The common bile duct and intrahepatic bile ducts were dilated. No obvious metastases were seen in other viscera or the lung fields, and intraperitoneal lymph nodes were not enlarged.

Based on imaging studies, we considered a duodenal sarcoma with bleeding and necrosis. Due to gastrointestinal obstruction and bleeding, urgent surgical resection was recommended to the patient and undertaken. A pylorus-preserving pancreaticoduodenectomy with partial resection of the inferior vena cava and right hemicolectomy were performed. The patient developed delayed gastric emptying and was discharged 22 days postoperatively.

Pathological examination of the pancreaticoduodenectomy specimen demonstrated a pale tan-colored tumor in the duodenum, measuring approximately 16 × 8 × 4 cm, accompanied by hemorrhage and necrosis, and invading into the serosa of the adjacent colon. The tumor invasion breached through the mucosa of the duodenum, extending to the duodenal papilla, but the common bile duct and the pancreas were uninvolved. Surgical margins were negative for tumor invasion.

**Fig. 3:**
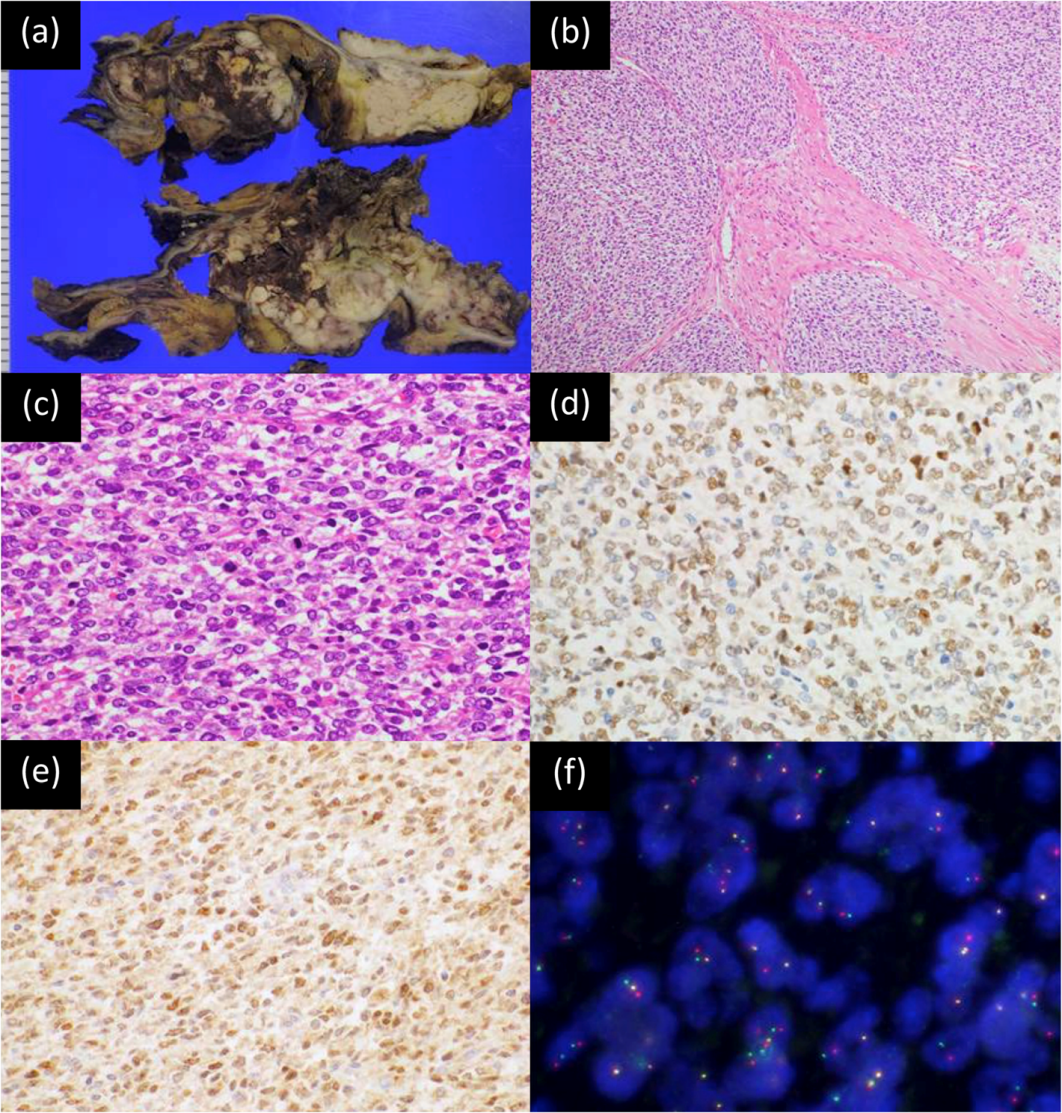
Pathological findings of the CIC-rearranged sarcoma. **a** Cut surface of the pancreaticoduodenectomy specimen shows a pale tan-colored tumor in the duodenum accompanied by hemorrhage and necrosis. **b** A low-power photomicrograph shows focal lobulation within a fibrotic stroma (20x). **c** A high-power photomicrograph reveals proliferation of round neoplastic cells with mild pleomorphism and focally prominent nucleoli (hematoxylin and eosin stain, 40x). **d** Neoplastic cells are positive for WT1 (immunohistochemistry, 40x). **e** Neoplastic cells are positive for ETV4 (immunohistochemistry, 40x). **f** Fluorescence in situ hybridization using a CIC break-apart probe set demonstrates CIC rearrangement, showing 5′ green and 3′ red abnormal split signals (100x)

Histologically, the tumor consisted of solid nodules of relatively uniform and round neoplastic cells (Fig. 3). Focal necrosis and lobulation with fibrous stromal septa were seen. The neoplastic cells harbored a mild degree of nuclear pleomorphism and focally prominent nucleoli accompanied by clear to slightly eosinophilic and scant cytoplasm, with a Ewing sarcoma-like appearance. The neoplastic cells were immunohistochemically positive for WT1 (diffuse), ETV4 (diffuse), vimentin (diffuse), CD99 (diffuse) and bcl-2 (diffuse and weak), but negative for epithelial membrane antigen, broad cytokeratins (AE1/AE3), low molecular weight cytokeratins (CAM5.2), CD34, CD45, CD56, CD117, DOG-1, S-100 protein, aSMA, desmin, myogenin, myoD1, chromogranin A, synaptophysin and D2–40. Fluorescence in situ hybridization (FISH) using a CIC break-apart probe set demonstrated CIC rearrangement. Thus, the final diagnosis of a CIC-rearranged sarcoma was made.

**Fig. 4:**
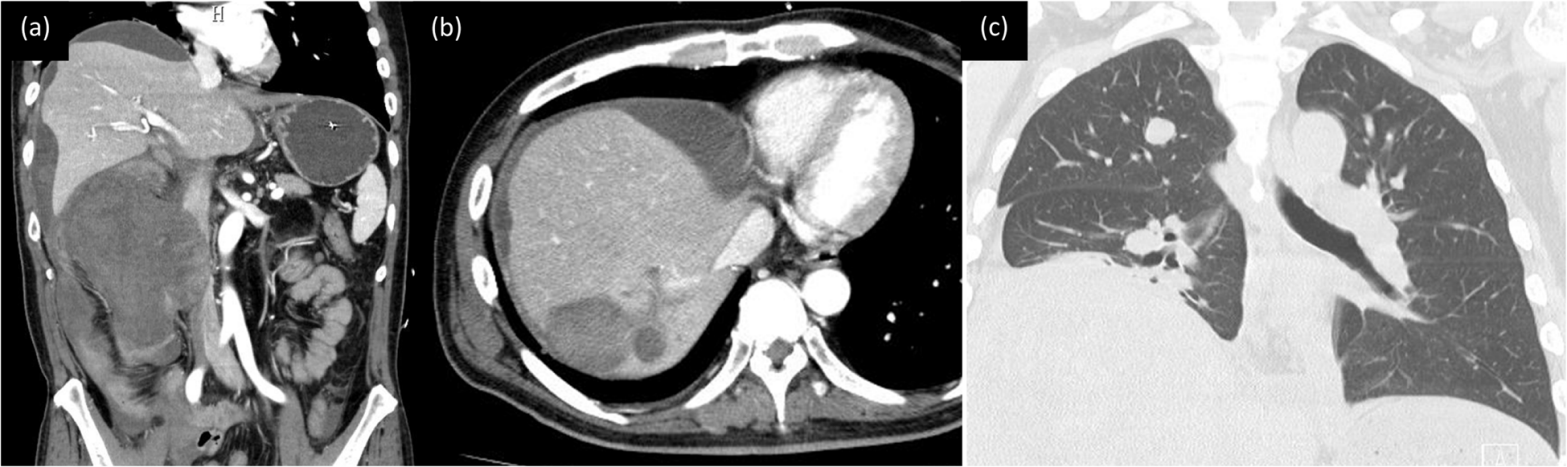
**a**-**c** Computed tomography scan shows a greater than 16 cm recurrent tumor with distant metastases in the posterior segment of the liver and the lingula of the lung

Two months postoperatively, the patient presented with low back pain. CT scan showed a new 16 cm tumor at the resection site and new lesions in the posterior segment of the liver and lingula of the lung (Fig. 4). This recurrence of the CIC-rearranged sarcoma progressed with rapid deterioration of the patient’s status. We provided palliative care, and the patient died 3 months postoperatively.

## Discussion and conclusions

This is the first report of a radical resection of a CIC-rearranged sarcoma of the duodenum diagnosed by a molecular genetic search. To control gastrointestinal bleeding from the tumor and resect the tumor completely, a pylorus-preserving pancreaticoduodenectomy with partial resection of the inferior vena cava and right hemicolectomy were performed.

Ewing sarcoma was first reported in 1921 as a small round cell tumor originating in bone by Ewing [2]. Ewing sarcoma occurs frequently in children or young adults and is a typical small round cell tumor known to express the EWSR-ETS fusion. Until now, small round cell sarcomas with a histologic appearance similar to Ewing sarcoma but without the EWSR-ETS fusion gene or translocation have been called Ewing-like sarcoma. Ewing-like sarcoma shares overlapping morphologic and clinical features with Ewing sarcoma. In recent years, CIC and BCL6 corepressor gene (BCOR) that are the most commonly rearrangement gene in Ewing-like sarcoma have been discovered and are being recognized as a separate disease.

CIC-rearranged sarcoma was first reported in 2006 as Ewing-like sarcoma with CIC-double homeobox protein 4 gene (DUX4) rearrangement by Kawamura-Saito [3]. The CIC gene present on chromosome 19 acts as a strong transcriptional activator by fusion with the DUX4 gene present on chromosome 4 or 10, causing a t (4; 19) or t (10; 19) translocation. The CIC-DUX4 fusion gene has been found to enhance the expression of EST variant 1/4/5 (ETV1/4/5). The DUX4 gene has been elucidated as the most common fusion gene with the CIC gene, but the existence of forkhead box O4 (FOXO4) gene and other fusion genes that have not yet been elucidated have also been suggested [4–7]. Therefore, Ewing-like sarcoma with this CIC gene rearrangement is collectively called CIC-rearranged sarcoma. Although CIC-rearranged sarcoma is extremely rare, it has recently been shown to exhibit different clinical, histological, and immunohistochemical features from Ewing sarcoma.

CIC-rearranged sarcoma affects a wide age range (6–81 years), but most commonly present in young adults (mean age, 30 years), and slightly more common in males than females. Nearly 90% of cases arise in soft tissues, occur equally in the extremities and trunk/pelvis, and arise in visceral organs is approximately 10%. Though Ewing sarcoma arises commonly in bone, most CIC-rearranged sarcoma arises in soft tissue and bone development is less than 5% [8]. Very rare cases have been reported in deep organs such as the gastrointestinal tract, kidney, and brain [4, 6, 7, 9].

Macroscopic features show a soft white-yellow tumor, often having a lobulated structure or extensive hemorrhage and necrosis. Although variable in size, gross features are generally similar to Ewing sarcoma. Microscopic features also resemble Ewing sarcoma in that they are composed of sheets of medium-sized round cells with indistinct cell borders. However, in various proportions, the pleomorphic tumor cells, spindle cells (approximately 10% of CIC-rearranged sarcoma), tumor cells with distinct nucleoli appeared and lobulated or myxomatous backgrounds are often observed, which are rarely observed in Ewing sarcoma [4–7, 10]. In addition, several reports showed that CIC-rearranged sarcoma tended to have a greater amount of eosinophilic cytoplasm and occasional clear cell change compared with conventional Ewing Sarcoma, and lack any evidence of neuroectodermal differentiation such as Homer Wright rosettes [8]. In rare cases, appearance of a minor component of rhabdoid or plasmacytoid morphology has been reported [5, 7, 10].

In an immunohistochemical expression pattern, most CIC-rearranged sarcomas exhibit only focal expression of CD99, which is strongly positive in the tumor cell membrane in more than 95% of Ewing sarcomas, and a small subset are completely negative [5, 7]. Nuclear EST variant transcription factor 4 gene (ETV4) and Wilms tumor suppressor gene (WT1) expression is seen in more than 90% of CIC-rearranged sarcomas whereas Ewing sarcoma, BCOR-rearranged sarcoma, and poorly differentiated synovial sarcoma are negative [5, 7, 11, 12]. CIC-rearranged sarcoma is also commonly positive for calretinin (approximately 70%), resulting in immunohistochemical overlap with mesothelioma [5]. In limited cases, focal desmin and cytokeratin AE1/AE3 and S100 expressed [4, 5, 7, 8]. Several neuroendocrine markers and some cell surface markers used for diagnosing lymphoma are generally negative [4, 5]. Some reports showed that NK2 homeobox 2 gene (NKX2.2) that is negative in most CIC-rearranged sarcomas is useful for diagnosis. NKX2.2 is a member of the NK2 family of transcription factors that determine development and differentiation, and is thought to be involved in Ewing sarcoma tumorigenesis when expressed in conjunction with Ewing sarcoma specific Ewing sarcoma region friend leukemia virus integration 1 (EWSR-FLI1) [12, 13]. Recently, diffuse MYC proto-oncogene (MYC) immunohistochemical expression has also been reported [14]. However, at present, a molecular genetic search is essential for a definitive diagnosis of CIC-rearranged sarcoma. In this case, both WT1 and ETV4 were positive and the tumor had a rearrangement of the CIC gene in a molecular genetic search with fluorescence in situ hybridization (FISH).

The only effective treatment for the CIC-rearranged sarcoma is complete resection. This patient suffered from gastrointestinal obstruction and gastrointestinal bleeding. The patient had been generally deteriorating and we regarded this as an oncologic emergency. We tried to perform complete resection of the tumor because of the necessity of the tumor resection for controlling gastrointestinal bleeding and this locally advanced duodenal tumor presumed to be highly malignant had not yet metastasized. Retrospectively this surgical strategy seemed to be appropriate treatment for a CIC-rearranged sarcoma. Pancreaticoduodenectomy is a technically demanding and complex procedure with a relatively high rate of complications and morbidity despite refinement of preoperative management and surgical technique [15]. Some authors propose a staged reconstruction to minimize the complications for emergent pancreaticoduodenectomy [16]. In this patient, we performed a primary reconstruction because the patient was young and his preoperative general condition seemed reasonable. However, in emergent pancreaticoduodenectomy, it is necessary to judge carefully and comprehensively whether to reconstruct primary or not by evaluating the patient’s general condition, vital signs, operative time and blood loss.

CIC-rearranged sarcoma has a typically poorer prognosis than Ewing sarcoma and frequently metastasizes. Most metastases are reported to the lung, and metastases to the liver, brain, lymph nodes, pleura, thyroid, bone, and soft tissues also have been reported [4, 5, 7]. Although chemotherapy is relatively effective in Ewing sarcoma, CIC-rearranged sarcoma is resistant to chemotherapy and often follows a more aggressive clinical course. Even in this patient, the tumor had advanced local progression, and the patient developed early local recurrence and distant metastases to the lung and liver and died despite radical resection.

In conclusions, although the definitive treatment for CIC-rearranged sarcomas is resection, CIC-rearranged sarcomas are highly malignant with a poor prognosis even after radical resection. The accumulation of further cases is desirable to establish optimal treatment strategies.

## Data Availability

The datasets used and/or analysed during the current study are available from the corresponding author on reasonable request.

## Abbreviations

CT: Computed tomography

## References

[1] Fletcher CDM, Bridge JA, Hogendoorn PCW, Mertens F (2013). WHO Classification of Tumours of Soft Tissue and Bone.

[2] J. E (1921). Diffuse endothelioma of bone. Proc New York Pathol Soc.

[3] Kawamura-Saito M, Yamazaki Y, Kaneko K, Kawaguchi N, Kanda H, Mukai H, Gotoh T, Motoi T, Fukayama M, Aburatani H (2006). Fusion between CIC and DUX4 up-regulates PEA3 family genes in Ewing-like sarcomas with t(4;19)(q35;q13) translocation. Hum Mol Genet.

[4] Italiano A, Sung YS, Zhang L, Singer S, Maki RG, Coindre JM, Antonescu CR (2012). High prevalence of CIC fusion with double-homeobox (DUX4) transcription factors in EWSR1-negative undifferentiated small blue round cell sarcomas. Genes Chromosom Cancer.

[5] Yoshida A, Goto K, Kodaira M, Kobayashi E, Kawamoto H, Mori T, Yoshimoto S, Endo O, Kodama N, Kushima R (2016). CIC-rearranged sarcomas: a study of 20 cases and comparisons with Ewing sarcomas. Am J Surg Pathol.

[6] Le Guellec S, Velasco V, Pérot G, Watson S, Tirode F, Coindre JM (2016). ETV4 is a useful marker for the diagnosis of CIC-rearranged undifferentiated round-cell sarcomas: a study of 127 cases including mimicking lesions. Mod Pathol.

[7] Antonescu CR, Owosho AA, Zhang L, Chen S, Deniz K, Huryn JM, Kao YC, Huang SC, Singer S, Tap W (2017). Sarcomas with CIC-rearrangements are a distinct pathologic entity with aggressive outcome: a Clinicopathologic and molecular study of 115 cases. Am J Surg Pathol.

[8] Carter CS, Patel RM (2019). Important recently characterized non-Ewing small round cell tumors. Surg Pathol Clin.

[9] Bielle F, Zanello M, Guillemot D, Gil-Delgado M, Bertrand A, Boch AL, Fréneaux P, Mokhtari K (2014). Unusual primary cerebral localization of a CIC-DUX4 translocation tumor of the Ewing sarcoma family. Acta Neuropathol.

[10] Gambarotti M, Benini S, Gamberi G, Cocchi S, Palmerini E, Sbaraglia M, Donati D, Picci P, Vanel D, Ferrari S (2016). CIC-DUX4 fusion-positive round-cell sarcomas of soft tissue and bone: a single-institution morphological and molecular analysis of seven cases. Histopathology.

[11] Sugita S, Arai Y, Tonooka A, Hama N, Totoki Y, Fujii T, Aoyama T, Asanuma H, Tsukahara T, Kaya M (2014). A novel CIC-FOXO4 gene fusion in undifferentiated small round cell sarcoma: a genetically distinct variant of Ewing-like sarcoma. Am J Surg Pathol.

[12] Hung YP, Fletcher CD, Hornick JL (2016). Evaluation of ETV4 and WT1 expression in CIC-rearranged sarcomas and histologic mimics. Mod Pathol.

[13] Yoshida A, Sekine S, Tsuta K, Fukayama M, Furuta K, Tsuda H (2012). NKX2.2 is a useful immunohistochemical marker for Ewing sarcoma. Am J Surg Pathol.

[14] Smith SC, Buehler D, Choi EY, McHugh JB, Rubin BP, Billings SD, Balzer B, Thomas DG, Lucas DR, Goldblum JR (2015). CIC-DUX sarcomas demonstrate frequent MYC amplification and ETS-family transcription factor expression. Mod Pathol.

[15] Tsai CY, Lai BR, Wang SY, Liao CH, Liu YY, Kang SC, Yeh CN, Jan YY, Yeh TS (2017). The impact of preoperative etiology on emergent pancreaticoduodenectomy for non-traumatic patients. World J Emerg Surg.

[16] Miyagawa S, Makuuchi M, Kawasaki S, Ogiwara M (1994). Second-stage pancreatojejunostomy following pancreatoduodenectomy in high-risk patients. Am J Surg.

